# In vitro antileishmanial and cytotoxicity activities of essential oils from *Haplophyllum tuberculatum* A. Juss leaves, stems and aerial parts

**DOI:** 10.1186/s12906-018-2128-6

**Published:** 2018-02-14

**Authors:** Assia Hamdi, Joanne Bero, Claire Beaufay, Guido Flamini, Zohra Marzouk, Yvan Vander Heyden, Joelle Quetin-Leclercq

**Affiliations:** 1 Department of Analytical Chemistry, Applied Chemometrics and Molecular Modelling (FABI), Center for Pharmaceutical Research (CePhaR), Vrije Universiteit Brussel (VUB), Laarbeeklaan 103, 1090 Brussels, Belgium; 2 Pharmacognosy Research Group (GNOS), Louvain Drug Research Institute (LDRI), Université catholique de Louvain (UCL), B1.72.03 Av. E. Mounier 72, B-1200 Bruxelles, Belgium; 3 Dipartimento di Scienze Farmaceutiche, Sede Chimica Bioorganica e Biofarmacia, Università di Pisa, Via Bonanno 33, 56126 Pisa, Italy; 4 Laboratoire de Développement Chimique Galénique et Pharmacologique des Médicaments. Faculté de Pharmacie, Université de Monastir, 5000 Monastir, Tunisia

**Keywords:** *Haplophyllum tuberculatum*, Essential oils, Limonene, Anti-leishmanial, Cytotoxicity

## Abstract

**Background:**

Plants used for traditional medicine produce diverse and complex secondary metabolites exhibiting various medicinal properties. The medicinal plant *Haplophyllum tuberculatum* is used by native people against malaria and parasitic infections.

**Methods:**

In this study and in order to contribute for the search of new natural drugs for leishmaniasis, the essential oils of *H. tuberculatum* leaves, stems and aerial parts (leaves+stems) collected in two different periods, 2013 and 2015, and their components by GC/FID and GC/MS analyses were investigated. Those collected in 2013 were also re-analyzed two years later. The extracted oils were screened in vitro for anti-leishmanial activity on *Leishmania mexicana mexicana* (*L.m.m.*) promastigotes and cytotoxicity on the Chinese Hamster Ovary (CHO) cell line. Limonene (1.5 – 8%), its isomers (*R*- (+)-limonene and *S*-(-)-limonene), linalool and octanol were also tested.

**Results:**

Results showed that the chemical composition varied according to the year of collection. Though major compounds remain almost the same, qualitative and quantitative variations in the composition of the EOs can be observed between the two years of collection, with some minor compounds identified only in one type of samples. Variation in the composition were also observed in the re-analyzed volatile oils, showing stability concerns. The essential oils and *R*-(+)-limonene showed moderate anti-leishmanial activity. Their IC_50_ range from 6.48 to 50.28 μg/ml. Cytotoxicity assays for theses volatile extracts, *R*- (+)-limonene and *S*- (-)-limonene on CHO cells showed relatively potent cytotoxicity with a selectivity index <10. Their CC_50_ range from 27.79 to 82.56 μg/ml.

**Conclusions:**

The findings of the present study demonstrated that *H. tuberculatum* might not be considered as a natural source for production of new anti-leishmanial agents without further analyzing its eventual in vivo toxicity as well as that of major pure compounds.

## Background

Leishmaniasis are parasitic diseases. More than 20 *Leishmania* species have about 70 natural reservoir hosts and vectors including animals, such as dogs or rodents, and human bodies, and are transmitted by more than 90 sand fly species. These diseases are considered as a serious health concern which are occurring in Africa, Asia, Southern Europe and Latin America. Their prevalence rate was estimated from 900 000 to 1.3 million new cases and from 20 000 to 30 000 deaths annually. The parasite may survive for decades in asymptomatic infected people and can also be transmitted directly from person to person. Many factors can increase leishmaniasis like malnutrition, migration, poor housing, a weak immune system, environmental and climatic changes such as deforestation and temperature variation and lack of financial resources [1–3]. In the absence of effective and sustainable vaccines, its control is still considered as a major public health problem [4]. The chemotherapy and the drugs based on pentavalent antimonials as sodium stibugluconate (Pentostam®) and meglumine antimoniate (Glucantime®) are the current means of treatment [5]. Therefore, the required long-term treatment, toxicity, grave side-effects, pain, cost, drug resistance associated with treatment failures point out the crucial need for new agents in the treatment of leishmaniasis [4, 6].

Natural products traditionally used in folk medicine to treat several diseases are analyzed worldwide. In this regard, complex mixtures or purified compounds obtained from organic/aqueous extracts and essential oils of roots, stems, leaves, flowers, fruits and spices are various sources of diverse bioactive natural constituents [7–9]. The investigations showed that wild and cultivated plants exhibited various pharmacological activities such as antimicrobial, anticancer, anti-inflammatory, antiparasitic including anti-leishmanial ones [10]. Moreover, some plants essential oils and their main constituents displayed anti-leishmanial activities [6].

*Haplophyllum tuberculatum* A. Juss is a perennial herb, belonging to the rutaceae family, native of North Africa and other areas of the Middle East. The aerial part of this plant is used as laxative, to cure gastro-intestinal affections, intermittent fevers, rheumatisms [11], malaria, gynecological disorders and renal disorders [12]. Moreover, reviews have reported *H. tuberculatum* as having nematicidal [13, 14], hepatoprotective [15], antiplasmodial [16, 17] and insecticidal activities [18]. Previous studies have also shown that *H. tuberculatum* is rich in volatile oils. Furthermore, these essential oils exhibited nematicidal [13], antimicrobial [19, 20], acetylcholinesterase inhibition and repellent activities [20].

The main aim of the present study is to analyze the volatile oils of different *H. tuberculatum* parts collected during two different seasons, to evaluate their anti-leishmanial effects against *L. mexicana mexicana* promastigote forms and their cytotoxic activities against CHO cells in an in vitro model. In order to relate the oils bioactivities to pure components ones, a major compound, limonene, and two minor ones, octanol and linalool, were tested in vitro. The other identified major compounds, *cis*-p-menth-2-en-1-ol, *trans*-p-menth-2-en-1-ol, *cis*-piperitol are not commercially available.

## Methods

### Plant material

Plant material from the *H. tuberculatum* Forssk. (A. Juss) species was collected at the end of December 2012 and May 2015 from Beni Ghzayel, Medenine, an arid region in Tunisia (33°21′17″ North 10°30′19″ East). The herbarium specimens were authenticated with their morphological and anatomical features in the Botany Department, Faculty of Pharmacy Monastir Tunisia and Botany Department of Faculty of Sciences Sfax Tunisia, according to the flora of Tunisia [21] and a voucher specimen (H.t-01.03) was deposited in the Biological Laboratory of the Faculty of Pharmacy of Monastir. The leaves, stems and roots were cut into small pieces and weighed before extraction of volatile compounds.

### Extraction of essential oils

About 100 g of fresh plant parts (leaves and/or stems or roots) was subjected to a 3 h hydrodistillation with 500 ml of distilled water using a Clevenger-type apparatus (Clevenger, 1928). The leaves (L), stems (S), and leaves+stems (LS) essential oils (EO) obtained were separated from the distilled water and dried on anhydrous sodium sulphate. No oil was obtained from the roots part. The volatile extracts were stored in sealed glass vials at 4-5 °C.

### Analytical GC-FID and GC-MS

Three analyzes were done to identify the differential components in the three oils. Analyze 1 (Anlz1) referred to the oils extracted in 2013 and analyzed in 2014. The oils samples were re-analyzed in 2016, mentioned as Analyze 2 (Anlz2), in order to study the stability of the components. The third one (Anlz3) referred to samples extracted in 2015 and analyzed in 2016.

### GC conditions

Gas chromatography (GC) analyses were carried out with an HP-5890 Series II instrument equipped with HP-WAX and HP-5 capillary columns (30 m×0.25 mm, 0.25 μm film thickness), working with the following temperature program: 60 °C for 10 min, ramp of 5 °C/min up to 220 °C; injector and detector temperatures 250 °C; carrier gas was helium (2 mL/min); detector dual FID; split ratio 1:30; injection of 0.5 μL (10 % hexane solution). Gas chromatography-electron impact mass spectroscopy analyses were performed with a Varian CP-3800 gas chromatograph, equipped with a HP-5 capillary column (30 m×0.25 mm; coating thickness 0.25 μm) and a Varian Saturn 2000 ion trap mass detector. Analytical conditions were as follows: injector and transfer line temperatures 220 and 240 °C, respectively; oven temperature programmed from 60 to 240 °C at 3 °C/min; carrier gas helium at 1 ml/min; injection of 0.2 μL (10 % hexane solution); split ratio 1:30 [22–27].

### Identification and quantification of oils components

The identification of the constituents was based on comparison of retention times with those of reference data or pure compounds based on their LRIs with the series of n-hydrocarbons. Moreover, identification of compounds also used gas chromatography-chemical ionization mass spectrometry, using methanol as the chemical ionizing gas [22–27] and computer matching against commercial (NIST 98 and ADAMS) and home-made library mass spectra (built up from pure substances and components of known oils and mass spectra literature data). The identified constituents were quantified by the normalization procedure using FID data.

### Standards compounds

Octanol (for synthesis, Darmstadt, Germany, Merck), linalool (Buchs, Switzerland, Fluka), *R*- (+)-Limonen and *S*- (-)-Limonen (96%, Steinheim, Germany, Sigma-Aldrich) were provided by commercial suppliers and dissolved in dimethyl sulfoxide (DMSO) (Darmstadt, Germany, Merck). MTT, amphotericin B and campthotecin came from Sigma-Aldrich (Steinheim, Germany) and Alamar Blue were provided by Thermo Fisher Scientific (Aalst, Belgium).

### Anti-leishmanial activity

The in vitro anti-leishmanial effects of essential oils from the leaves (LEO), stems (SEO) and the aerial parts of *H. tuberculatum* (LSEO) were investigated on *Leishmania mexicana mexicana* (*L.m.m.*) as described by [28]. The solutions were prepared in DMSO at 20 mg/ml. Parasites in the logarithmic growth phase were seeded in 96-well culture plates. Essential oils and compounds were tested in eight serial threefold dilutions (0.05-100 μg/ml, 2 wells/concentration). Amphotericin B was used as positive control with an initial concentration of 1μg/ml. The plate was kept at 28°C and after 72 h of incubation, *Leishmania* viability was calculated by quantification of Alamar Blue fluorescence (10 μl diluted two times in PBS/well incubated 4h), using an excitation wavelength of 530 nm and emission one of 590 nm on a SpectraMax M2e (Molecular Devices) spectrophotometer. Assays were performed in triplicate to calculate IC_50_.

### Cytotoxicity assay and selectivity index

A tetrazolium salt colorimetric method has been used to determine survival of Chinese Hamster Ovary (CHO) as described by Bero et al., 2009, with minor modifications [29]. The MTT (3-[4,5-dimethylthiazol-2-yl]-2,5 diphenyl tetrazolium bromide) assay is based on the conversion of MTT into formazan crystals by mitochondrial dehydrogenase in living cells. The solutions of essential oils were prepared in DMSO at 20 mg/ml. The essential oils and compounds were firstly diluted in medium in eight serial threefold dilutions (0.5-100 μg/ml, 3 wells/concentration) in 96-well microtiter plate. Secondly, 20 μl of diluted solutions were transferred to 180 μl of 5 10^3^ cells previously incubated during 24 h. Finally, after 72 h of incubation, the medium was replaced by a 10% MTT solution (3 mg/ml in PBS) in fresh medium and after 45 min, the medium was replaced again by 100μl of DMSO added to solubilize formed formazan crystals. Absorbance was recorded at 570 and 620 nm on a SpectraMax M2e (Molecular Devices) spectrophotometer. Camptothecin was used as positive control at an initial concentration of 25μg/ml. Assays were performed in duplicate to calculate the CC_50_. The selectivity index (SI), expressed by the CC_50_/IC_50_ ratio [30], defines the balance between cytotoxicity and anti-leishmanial activity. If the SI value is higher than 10, treatment is considered as safe for the cells (CHO) at the therapeutic concentration [31, 32].

### Statistical analysis

The IC_50_ and CC_50_ of the bioassays were calculated by the least square ordinary fit method based on a sigmoidal curve. Statistical analysis was performed using Microsoft Excel as the average ± SE for triplicates and duplicates of the anti-leishmanial and cytotoxicity assay, respectively. Data analysis was carried out by using SPSS statistical package version 16.0. Differences between the tests were analyzed by Duncan-test. *P*<0.05 was considered as statistically significant.

## Results

### Chemical profiles of the oils

The comparison of the data obtained for the same essential oil (EO) obtained from *H. tuberculatum* analyzed in 2014 and re-analyzed in 2016 showed qualitative and quantitative variations. Moreover, different chemical profiles were also observed between the EO extracted in 2013 and in 2015 (Table 1).

**Table 1:** Chemical composition of the essential oil of *Haplophyllum tuberculatum* vegetal parts

		EO 2013 (Anlz1)			EO 2013 (Anlz2)			EO 2015 (Anlz3)		
Constituents	l.r.i.^a^	LEO	SEO	LSEO	LEO	SEO	LSEO	LEO	SEO	LSEO
ethyl isovalerate	854	-	-	-	-	-	-	-	0.3	-
tricyclene	928	-	-	-	-	-	0.1	-	-	0.2
α-thujene	933	-	-	-	-	-	-	-	-	0.2
α-pinene	941	2.9	4.6	3.9	1.4	3.4	4.6	2.1	4.6	7.2
camphene	955	0.9	3.0	2.5	0.6	2.1	2.8	0.8	2.5	3.2
sabinene	977	0.4	-	0.7	0.2	0.3	0.6	0.3	0.5	0.9
β-pinene	981	1.1	3.4	2.9	0.7	2.5	3.0	0.9	3.5	3.9
myrcene	993	3.3	0.8	1.3	0.7	0.4	0.9	1.0	2.1	2.6
octanal	1002	-	-	-	-	-	0.3	-	-	-
α-phellandrene	1006	0.4	-	0.7	-	-	-	-	2.8	3.8
α-terpinene	1020	-	-	0.3	-	-	-	-	-	-
*p*-cymene	1028	1.9	2.0	3.2	0.7	-	2.3	0.9	0.8	2.2
limonene	1032	8.1	2.3	5.2	2.0	1.5	2.9	2.8	6.4	9.4
*(E)*-β-ocimene	1052	-	-	-	-	-	-	-	-	0.2
*(E)*-2-octen-1-ol	1071	0.4	-	0.7	0.6	0.9	0.7	0.6	0.6	0.4
1-octanol	1074	0.3	1.0	0.5	-	0.3	-	-	-	-
1-nonen-3-ol	1088	0.7	1.3	-	0.6	1.1	2.0	0.6	0.6	1.1
2-nonanone	1093	-	-	0.3	-	-	0.3	-	-	-
2-methylbutyl 2-methylbutanoate	1101	-	-	-	-	-	-	-	0.3	-
linalool	1101	0.7	-	0.9	0.5	-	0.7	0.4	-	0.7
isopentyl 2-methylbutanoate	1103	0.6	-	0.8	0.7	0.6	0.7	0.8	-	-
isopentyl isovalerate	1104	0.4	0.6	0.6	-	-	0.4	-	2.0	1.0
pentyl isovalerate	1106	-	-	-	0.4	0.5	0.7	0.4	1.5	0.7
*cis-p*-menth-2-en-1-ol	1123	16.8	12.4	8.7	19.9	12.7	13.7	21.3	15.9	16.0
*trans-p*-menth-2-en-1-ol	1142	16.2	11.2	8.2	17.8	11.2	12.2	18.8	11.8	12.6
pinocarvone	1164	-	-	0.3	-	-	0.3	-	-	-
ethyl benzoate	1172	-	-	-	-	-	-	-	0.5	-
1-nonanol	1173	-	-	-	0.2	-	-	-	-	-
4-terpineol	1179	0.4	-	0.8	-	0.7	0.6	-	0.5	0.4
cryptone	1186	-	-	4.5	1.7	3.8	3.3	1.5	1.4	1.4
1-dodecene	1193	-	-	-	-	0.8	-	-	-	-
*cis*-piperitol	1195	4.9	4.0	2.6	5.9	3.2	2.8	5.6	4.1	3.1
*trans*-piperitol	1207	12.1	9.1	5.5	7.9	3.2	-	8.0	4.2	-
1-octyl acetate	1213	5.4	7.4	8.8	10.5	11.0	15.2	9.9	8.1	12.4
3-methyl-3-hexen-1-yl butanoate	1235	0.8	-	-	-	-	-	-	-	-
*(Z)*-3-hexenyl isovalerate	1238	-	-	-	0.2	-	-	-	-	-
2-nonyl acetate	1243	-	-	-	-	-	1.0	0.7	0.3	0.5
hexyl isovalerate	1243	-	-	-	0.6	0.6	-	-	-	-
cumin aldehyde	1244	-	0.9	-	0.2	0.3	0.4	-	-	-
piperitone	1254	6.7	8.5	9.1	8.1	8.0	9.6	7.5	6.8	5.2
1-decanol	1272	-	-	-	1.5	-	-	1.5	-	-
*(Z)*-3-tridecene	1282	-	1.6	2.0	-	-	-	-	-	-
isobornyl acetate	1287	2.0	13.8	7.8	3.0	15.0	9.8	2.8	9.8	7.3
2-undecanone	1293	0.4	1.0	0.9	0.4	0.6	0.5	0.4	0.5	0.2
2-undecanol	1304	-	-	0.6	-	-	-	-	-	-
*neo-iso*-isopulegol acetate	1310	-	-	0.7	-	-	-	-	-	-
myrtenyl acetate	1327	-	-	0.3	-	0.7	-	-	-	-
3-oxo-*p*-menth-1-en-7-al	1334	-	-	0.3	-	-	-	-	-	-
*trans*-piperitol acetate	1343	-	0.9	1.0	0.6	0.9	0.9	0.6	0.6	0.4
neryl acetate	1365	0.5	0.7	0.5	0.6	0.6	0.4	0.6	0.6	0.3
geranyl acetate	1383	-	-	-	0.3	0.2	-	-	-	-
1-decyl acetate	1411	-	-	-	0.5	0.5	0.3	0.5	0.4	-
1-decanol acetate	1412	0.4	-	0.8	-	-	-	-	-	-
1-octyl 2-methylbutanoate	1434	1.6	1.1	1.5	1.6	0.7	1.0	1.7	0.8	0.7
1-octyl isovalerate	1440	1.2	1.9	1.4	1.1	0.8	0.8	1.0	1.0	0.6
germacrene D	1482	-	-	-	-	-	-	-	0.7	-
*ar*-curcumene	1483	0.6	-	-	0.7	0.3	-	0.6	-	-
2-phenylethyl isovalerate	1490	-	-	-	0.2	-	-	-	-	-
valencene	1493	0.6	0.8	0.5	0.6	0.5	0.3	0.6	0.6	-
α-zingiberene	1496	-	-	-	-	-	-	-	0.3	-
β-sesquiphellandrene	1525	0.8	-	-	0.8	-	-	0.7	0.5	-
kessane	1526	-	-	-	-	0.3	-	-	-	-
tetradecanal	1612	-	-	-	0.4	-	-	0.3	-	-
α-cadinol	1654	-	-	-	0.3	0.3	-	-	0.3	-
γ-dodecalactone	1677	-	-	-	0.3	0.3	-	-	-	-
*cis*-14-*nor*-muurol-5-en-4-one	1687	-	-	-	0.3	-	-	-	-	-
pentadecanal	1716	0.9	-	-	1.9	0.3	-	1.5	0.3	-
Monoterpene hydrocarbons		19.0	16.1	20.7	6.3	10.2	17.2	8.8	23.2	33.8
Oxygenated monoterpenes		60.3	61.5	46.7	64.8	56.7	51.4	65.6	54.3	46.0
Sesquiterpene hydrocarbons		2.0	0.8	0.5	2.1	0.8	0.3	1.9	2.1	0.0
Oxygenated sesquiterpenes		-	-	-	0.6	0.6	0.0	0.0	0.3	0.0
Non-terpene derivatives		13.1	15.9	23.4	23.4	22.8	27.2	21.4	18.6	19.0
Total identified		94.4	94.3	91.3	97.2	91.1	96.1	97.7	98.5	98.8

^a^Linear retention indices (DB-5 column)

Analyze 1 (Anlz1): volatile oils extracted in 2013 and analyzed in 2014

Analyze 2 (Anlz2): volatile oils extracted in 2013 and re-analyzed in 2016

Analyze 3 (Anlz3): volatile oils extracted in 2015 and analyzed in 2016

The number of extraction repetitions: all EOs samples are extracted once

The main components present in the nine EO samples are α-pinene, limonene, *cis*-p-menth-2-en-1-ol, *trans*-p-menth-2-en-1-ol, *cis*-piperitol, 1-octyl acetate, piperitone, isobornyl acetate with variation in their area percentages. *Trans*-piperitol is also a major compound in most oils but it was not detected in both LSEO analyzed in 2016. The minor compounds found in all EOs are 2-undecanone, neryl acetate, 1-octyl 2-methylbutanoate, 1-octyl isovalerate. The amounts of myrcene and camphene vary between 0.8 and 3.3 % and 0.6 and 3.2 % respectively.

By comparing the EO analyzed in 2014 and re-analyzed in 2016, new compounds were detected in that of 2016 as tricyclene, octanal, pentyl isovalerate, 1-nonanol, 1-dodecene, *(Z)*-3-hexenyl isovalerate, 2-nonyl acetate, hexyl isovalerate, 1-decanol, geranyl acetate, 1-decyl acetate, 2-phenylisovalerate, kessane, tetradecanal, *α*-cadinol, γ-dodecalactone, *cis*-14-*nor*-muurol-5-en-4-one but as minor compounds. However, *α*-phellandrene, *α*-terpinene, 3-methyl-3-hexen-1-yl butanoate, *(Z)*-3-tridecene, 2-undecanol, *neo-iso*-isopulegol acetate, 3-oxo-*p*-menth-1-en-7-al, 1-decanol acetate disappeared in the Anlz2, but these compounds were minor in Anlz1 (Table 1).

The non-terpene derivatives increased in all oils. They were ranging from 13.1% to 23.4%, from 15.9% to 22.8 and from 23.4% to 27.2% for LEO, SEO and LSEO respectively. The oxygenated monoterpenes increased from 60% to 64.8%; from 46.7% to 51.5% for LEO and LSEO respectively and decreased from 61.5% to 56.7% for SEO, while the monoterpene hydrocarbons decreased in the EOs of all parts, from 19.0% to 6.3%; from 16.1 to 10.2% and from 20.7 to 17.2% for LEO, SEO and LSEO respectively.

On the other hand, new compounds were detected in the Anlz3 compared to Anlz1 as ethyl isovalerate, α-thujene, *(E)*-β-ocimene, 2-methylbutyl 2-methylbutanoate, ethyl benzoate, 1-decanol, 1-decyl acetate, germacrene D, α-zingiberene, tetradecanal, α-cadinol but some of them were present in Anlz 2. Moreover, some compounds were not detected in the Anlz3 as α-terpinene, 1-octanol, 2-nonanone, pinocarvone, 3-methyl-3-hexen-1-yl butanoate, cumin aldehyde, *(Z)*-3-tridecene, 2-undecanol, *neo-iso*-isopulegol acetate, myrtenyl acetate, 3-oxo-*p*-menth-1-en-7-al, 1-decanol acetate. When comparing Anlz 1 and Anlz 3, monoterpene hydrocarbons decreased from 19.0% to 8.8% for LEO; increased from 16.1 to 23.2% and from 20.7 to 33.8% for SEO and LSEO, respectively. The oxygenated monoterpenes increased from 60.3% to 65.6% for LEO, decreased from 61.5% to 54.3% and from 46.7% to 46.0% for SEO and LSEO respectively. The non-terpene derivatives increased from 13.1% to 21.4% from 15.9% to18.6% for LES and SEO respectively and decreased from 23.4% to 19.0% in LSEO.

Dissimilarity was noted in the Anlz2 and Anlz3. Eight compounds were detected in the Anlz3 but not in Anlz2: ethyl isovalerate, α-phellandrene, α-thujene, *(E)*-β-ocimene, 2-methylbutyl 2-methylbutanoate, ethyl benzoate, germacrene D, α-zingiberene. However, octanal, α-terpinene, 1-octanol, 2-nonanone, pinocarvone, 1-nonanol, 1-dodecene, 3-methyl-3-hexen-1-yl butanoate, *(Z)*-3-hexenyl isovalerate, hexyl isovalerate, cumin aldehyde, *(Z)*-3-tridecene, 2-undecanol, *neo-iso*-isopulegol acetate, myrtenyl acetate, 3-oxo-*p*-menth-1-en-7-al, geranyl acetate, 1-decanol acetate, 2-phenylethyl isovalerate, kessane, γ-dodecalactone, *cis*-14-*nor*-muurol-5-en-4-one were not identified in the Anlz3. The monoterpene hydrocarbons increased in the EO of all parts, from 6.3% to 8.8%; from 10.2 to 23.2% and from 17.2 to 33.8% for LEO, SEO and LSEO respectively. Moreover, the oxygenated monoterpenes increased from 64.8% to 65.6% for LEO, decreased from 56.7% to 54.3% and from 51.4% to 46.0% for SEO and LSEO respectively. However, the non-terpene derivatives decreased in all the oils. They were ranging from 23.4% to 21.4%, from 22.8% to 18.6 and from 27.2%to 19.0% for for LEO, SEO and LSEO respectively. In general, *H. tuberculatum* EO were characterized by high percentages of oxygenated monoterpenes followed by hydrocarbon monoterpenes and non-terpene derivatives.

### Anti-leishmanial activity

In this study, promastigotes of *L.m.m.* were incubated in the presence of various concentrations of essential oils and pure compounds. The cell viability was determined after 72 h using Alamar Blue assay. This is the first time that the anti-leishmanial activity of *H. tuberculanum* essential oils is shown with IC_50_ range from 6.48 to >100 μg/ml and dose-dependent responses (*P*≤0.05: Table 2).

**Table 2:** Anti-leishmanial (IC_50_ ±SD) and cytotoxic activity (CC_50_ ±SD) of essentials oils, some of their components and the positive controls

	Anti-leishmanial activity – promastigotes (*L. mexicana mexicana*)	Cytotoxicity -CHO	Selectivity index
Samples	IC_50_ (μg/ml) ± SD	CC_50_ (μg/ml) ± SD	CHO/*Lmm*
LEO 2013	16.69±0.35^c^	81.20±1.59^d^	4.86
SEO 2013	16.00±0.56^c^	49.64±8.56^c^	3.10
LSEO 2013	6.48±0.44^b^	27.79±3.73^b^	4.29
LEO 2015	19.44±1.00^c^	80.65±1.60^d^	4.15
SEO 2015	50.28±2.13^d^	82.56±5.51^d^	1.64
LSEO 2015	61.79±5.05^e^	79.06±0.80^d^	1.28
*R*- (+)-limonene	16.59±0.35^c^	29.65±0.78^b^	1.79
*S*- (-)-limonene	>100	75.89±0.88^d^	-
1-octanol	>100	>100	-
linalool	>100	>100	-
Amphotericin B	0.10±0.01^a^	nd	-
Camptothecin	nd	0.74±0.09^a^	-

The letters (a–e) indicate significant differences between the samples according to the Duncan Test (*p*<0.05)

The LEO 2013 and SEO 2013 had a similar effect while LSEO 2013 was the most active on *L.m.m.* (IC_50_ values: 16.69, 16.00 and 6.48 μg/ml, respectively). The LEO, SEO and LSEO, samples collected and extracted in 2015, showed IC_50_ values of 19.44, 50.28 and 61.79 μg/ml, respectively with a significant decrease in activity for SEO and LSEO 2015. Moreover, there is no significant difference between the IC_50_ of LEO 2013, SEO 2013 and that of LEO 2015 (*p* < 0.05).

### Cytotoxicity and selectivity index

Cytotoxic effects of *H. tuberculatum* oils were determined in CHO cells using MTT assay. The obtained finding indicated significant cytotoxicity of the essential oils and the tested pure compounds on CHO cells with CC_50_ range from 27.79 to >100 μg/ml (Table 2).

*R*-(+)-limonene (CC_50_ = 29.65 μg/ml) and LSEO 2013 (CC_50_ = 27.79 μg/ml) had the highest cytotoxic effect on cells compared to the other samples. LEO 2013, LEO 2015, SEO 2015, LSEO 2015 and *S*-(-)-limonene exhibited a similar low cytotoxic effect (*P* > 0.05).

The selectivity index (SI) for LEO extracted in 2013 and 2015 and LSEO 2013 were 4.86, 4.15 and 4.29, respectively, showing that they were less toxic on CHO cells than *Leishmania* parasites. However, in all cases, they are lower than 10, the minimum value defined for the selection of an anti-parasitic hit [31, 32]. Thus these oils could perhaps be used safely at therapeutic concentration in short term treatments but *R*- (+)-limonene (SI=1.79) cannot be considered as a drug candidate for the development of anti-leishmanial agents.

## Discussion

### Chemical profiles of the oils

Literature review showed variation between chemical compositions, depending on the location and stages of development of *H. tuberculatum*. Reports from Oman [19], Iran [33] and the United Arab Emirates [34] showed a difference between the major compounds. The comparison of the chemical composition from the different parts of our *H. tuberculatum* with that of *H. tuberculatum* from Oman showed that the main compounds varied [19]. Indeed, the main compounds of *H. tuberculatum* from Oman were beta-phellandrene (23.3%), limonene (12.6%), (Z)-beta-ocimene (12.3%), beta-caryophyllene (11.6%), myrcene (11.3%), and alpha-phellandrene (10.9%) [19]. Another study showed that the main constituents of the EO from the aerial parts were limonene (27.3%) and α-pinene (21.9%) [33]. Furthermore, the composition of the EO varied also according to the collecting season.

The analysis of the aerial parts of *H. tuberculatum* collected in May 1997 and 2001 from the United Arab Emirates showed that the major compounds were α-phellandrene (10.7–32.9%), β-caryophyllene (6.3–12.8%), β-pinene (7.6–8.0%), limonene (4.0–9.6%) and δ-3-carene (5.5–6.0%). However, the oil distilled from plants collected in April (1998) had as major components linalool (15.0%), linalyl acetate (10.6%), β-caryophyllene (9.7%) and α-terpineol (6.7%) [34].

Conversion and degradation reactions can be involved in these changes because of the metabolic relation between terpenoid biosynthesis and the effect of different factors such as temperature or light. This was already observed i.e. for laurel and fennel [35, 36]. The differences in composition observed between our results and those of the previous works might be related to the analyzed plant part (leaves, stems, leaves+stems and aerial part), the geographic origin of the populations, the ecological conditions in which they grow and also chemical instability or transformation, but may also suggest the existence of a new chemotype.

### Anti-leishmanial activity

This variability in anti-leishmanial activity may be explained by the qualitative and quantitative differences in chemical compositions of each essential oils. Indeed, literature showed that the chemical composition of essential oils and so their biological activities may be affected by the growing seasons. Some factors may be considered as responsible for these variations as temperature, rainfall and humidity which can affect plant metabolism and lead to composition differences [37].

Among the pure compounds, *R*-(+)-limonene was the most active one (IC_50_ = 16.59 μg/ml) compared to inactive *S*-(-)-limonene, linalool and 1-octanol (IC_50_ >100μg/ml). It can explain a part of the anti-leishmanial activity but other active compounds still remain to be identified. Kpadonou Kpoviessi et al., 2014 also, showed that *R*-(+)-limonene had an antitrypanosomal effect with IC_50_=4.24 ±2.27 μg/mL on *Trypanosoma brucei brucei* bloodstream forms [38]. As well, linalool was shown to have a strong inhibitory activity on *L. amazonensis* promastigotes and amastigotes with LD_50_ of 15.5 ng/ml and 4.3 ng/ml, respectively [39].

Some of the compounds present in our volatile oils were already tested for their anti-parasitic activities. Thus, the IC_50_ of alpha-pinene (55.3μg/mL; 4.1μg/mL), sabinene (126.6μg/mL; 17.7μg/mL), *β*-pinene (200.1μg/mL; 54.8μg/mL), *α*-phellandrene (32.8μg/mL; 9.2μg/mL), 4-terpineol (335.9μg/mL; 0.02μg/mL) were reported against promastigotes of *L. major* and the bloodstream forms of *T. brucei* (TC 221), respectively [40].

### Cytotoxicity and selectivity index

Both isomers, *R*-(+)-limonene and *S*-(-)-limonene, can account for at least a part of the cytotoxic activity of the EOs. Thus, once again, the cytotoxic variabilities of the samples can be related to the studied plant part and the season of collection [37].

The cytotoxicity of *R*-(+)-limonene was a little higher than that described by Kpoviessi et al. (2014) but the difference (factor less than 2) may be explained by the general biological variability of cells in culture. Moreover, our study showed that 1-octanol and linalool (CC_50_>100μg/ml) had no cytotoxicity on CHO cells.

Some studies analyzed the specific cellular targets of single components on the cells. Recent research demonstrated that linalool was not cytotoxic on some cell types (CC_50_ > 200 μg/ml): Vero, Macrophages, A-549, HeLa, HT-29 cells Saulo [41] but can inhibit mitochondrial complexes I and II, increase reactive oxygen species and inhibit the HepG2 cells viability (IC_50_=0.4 μM) [42].

Apoptosis induction was observed for linalool on MCF7 WT cells (IC_50_=0.62–0.79 μM) [43] and on HL-60 (IC_50_=49.53–127.14 μM) [44], limonene on K562 (IC_50_=ND) [45], HT-29 5 (IC_50_ >200 μg/mL) [46], alpha-pinene on U937 (IC_50_=ND) [47], pancreatic, mammary, and prostatic tumors (IC_50_=ND) [48].

No cytotoxicity was observed for 1-octanol. Published data on the cytotoxicity of other *H. tuberculatum* components showed a great activity variability according to used cell types. Alpha-pinene was cytotoxic on K562 (IC_50_=117.3μM) [49], HL-60 (IC_50_=2.5 μg/mL) [40], A-549 (IC_50_=183.2 μg/mL), HeLa (IC_50_=172.7 μg/mL), but not on Vero, macrophages and HT-29 [41]. Sabinene (IC_50_=23.6 μg/mL) and α-phellandrene (IC_50_= 26.9μg/mL) were cytotoxic on HL-60, respectively [40]. The cytotoxicity of β-pinene was studied on CHO and WI38 (IC_50_>50 μg/mL), HL-60 (IC_50_=29.6μg/mL) [40] and K562 (IC_50_=157.4μM) [49]. Myrcene showed an activity on HepG2 (IC_50_= 9.23μg/ml), B16-F12 (IC_50_=12.27μg/ml) [50], but not on Vera, macrophages, A 549, HT 29, CHO, PBMC and WI38 cells [41, 51]. *p*-Cymene exhibited a cytotoxic effect on B16-F12 (IC_50_=20.06 μg/mL) but not on CHO, WI38 [51], HepG2 and on PBMC [50]. 4-Terpineol demonstrated an effect on HL-60 (IC_50_=20.5μg/mL) [40] and on AE17 (IC_50_=0.02%; 0.01%), B16 (IC_50_= 0.05%; 0.04%), HF32 (IC_50_=0.1%; 0.1%) after 24 and 48h, respectively [52].

Literature data indicate that *H. tuberculatum* organic extracts possess a high cytotoxicity [53, 54]. This cytotoxicity may be explained by the cytotoxic lignans already identified in this species [55]. However, it cannot explain cytotoxicity of its essential oils which should not contain these lignans.

Previous studies showed that promastigotes cultures are a validated model for primary screening. Promastigotes are known to be less sensitive than amastigotes to drugs and allow a restricted selection of the most active samples before performing an intracellular amastigotes test [56]. However, as shown by the present results, the EOs have high cytotoxicity and the selectivity indices are not encouraging enough to proceed to the intracellular tests.

## Conclusions

Results showed that the chemical composition varied according to the year of collection, but that stabilities issues may also modify the composition n along the time, as shown by the variation observed in the re-analyzed volatile oils.

Our results showed that the chemical composition of the essential oils of different parts (leaves, stems and leaves+stems) from *H. tuberculatum* varied according the period of collection. The re-analyzed volatile oils indicated that stabilities issues may also modify their composition along the time. The tested essential oils and one of their major component (*R*-(+)-limonene) were biologically active against *L. mexicana mexicana* coupled to a cytotoxic effect on CHO in vitro.

However, as limonene is only present at concentration < 10%, other compounds and/or synergistic effects remain to be analyzed to explain the observed activities. Thus, the biological activities of the identified major compounds, *cis*-p-menth-2-en-1-ol, *trans*-p-menth-2-en-1-ol, *cis*-piperitol should be evaluated.

The results obtained in this study confirm the importance of chemical and biological investigations of essential oils but also toxicity risks. So in vitro studies are needed to assess the potential of these oils as anti-leishmanial agents and analyze deeper toxicity risks.

To our knowledge this is the first report of the anti-leishmanial and cytotoxic activity of the essential oils of *H. tuberculatum*.

## Abbreviations

CC_50_: Cytotoxic concentration of extract decreasing by 50% the number of viable cells
EO: Essential oil
IC_50_: Sample concentration inhibiting 50% of viable parasites
LEO/SEO/LSEO: Leaves/stems/leaves+stems essential oils
*L.m.m*: *Leishmania mexicana mexicana*
MTT: 3-[4,5-dimethylthiazol-2-yl]-2,5 diphenyl tetrazolium bromide
